# Role of noncoding RNAs and untranslated regions in cancer: A review

**DOI:** 10.1097/MD.0000000000030045

**Published:** 2022-08-19

**Authors:** Yiping Zhang, Meiwen Yang, Shulong Yang, Fenfang Hong

**Affiliations:** a Experimental Centre of Pathogen Biology, Nanchang University, Nanchang, China; b Queen Mary College, School of Medicine, Nanchang University, Nanchang, China; c Department of Surgery, Fuzhou Medical College, Nanchang University, Fuzhou, China; d Department of Physiology, Key Research Laboratory of Chronic Diseases, Fuzhou Medical College, Nanchang University, Fuzhou, China; e Department of Physiology, College of Medicine, Nanchang University, Nanchang, China.

**Keywords:** cancer, long non-coding RNAs, microRNAs, non-coding RNAs, untranslated regions

## Abstract

Cancer is one of the most prevalent diseases worldwide, and poses a threat to human health. Noncoding RNAs (ncRNAs) constitute most transcripts, but they cannot be translated into proteins. Studies have shown that ncRNAs can act as tumor suppressors or oncogenes. This review describes the role of several ncRNAs in various cancers, including microRNAs (miRNAs) such as the miR-34 family, let-7, miR-17-92 cluster, miR-210, and long noncoding RNAs (lncRNAs) such as HOX transcript antisense intergenic RNA (HOTAIR), Metastasis associated lung adenocarcinoma transcript 1 (MALAT1), H19, NF-κB-interacting lncRNA (NKILA), as well as circular RNAs (circRNAs) and untranslated regions (UTRs), highlighting their effects on cancer growth, invasion, metastasis, angiogenesis, and apoptosis. They function as tumor suppressors or oncogenes that interfere with different axes and pathways, including p53 and IL-6, which are involved in the progression of cancer. The characteristic expression of some ncRNAs in cancer also allows them to be used as biomarkers for early diagnosis and therapeutic candidates. There is a complex network of interactions between ncRNAs, with some lncRNAs and circRNAs acting as competitive endogenous RNAs (ceRNAs) to decoy miRNAs and repress their expression. The ceRNA network is a part of the ncRNA network and numerous ncRNAs work as nodes or hubs in the network, and disruption of their interactions can cause cancer development. Therefore, the balance and stabilization of this network are important for cancer diagnosis and treatment.

## 1. Introduction

Cancer is a constant threat to human health and a common health and safety issue facing humanity worldwide. In a statistical survey in the United States, cancer was considered the second leading cause of death after heart disease.^[1]^ They pose a serious threat to human health, and their diagnosis and treatment have therefore become a major issue to be addressed. Studies have found that noncoding RNAs (ncRNAs) play an important role in the development of cancer. ncRNAs account for approximately 98% of all RNAs and cannot encoded into proteins.^[2]^ There are 15 classes of ncRNAs that are classified into different groups, depending on the classification criteria. In terms of their functions, ncRNAs can be divided into two families: housekeeping ncRNAs and regulatory ncRNAs. Housekeeping ncRNAs, such as ribosomal RNAs (rRNAs), messenger RNAs (mRNAs) and transfer RNAs (tRNAs) involved in cellular genetic activities such as protein synthesis, RNA splicing, and RNA modification.^[3]^ Housekeeping ncRNAs act steadily in the production of proteins, and their roles have been extensively studied; therefore, they are not the subject of this review.

Regulatory ncRNAs act as regulators that participate in gene expression at the chromatin remodeling, transcriptional, and posttranscriptional levels, and function in signal transduction.^[4]^ Based on length, regulatory ncRNAs can be subclassified into two main groups: small ncRNAs (<200 nt) and long ncRNAs (>200 nt).^[3]^ Small ncRNAs include microRNAs (miRNAs), small interfering RNAs (siRNAs) and piwi-interacting RNAs (piRNAs). Among these, miRNAs are the most abundant and studied. Besides, some ncRNAs are variable in length, such as circular RNAs (circRNAs) and enhancer RNAs (eRNAs).^[5]^

ncRNAs do not encode proteins but are involved in many biological activities, particularly in gene regulation.^[6]^ In-depth studies of genes and disease mechanisms have revealed that abnormalities in ncRNAs can affect the development and progression of a variety of complex diseases, such as cancer. ncRNAs can affect cell differentiation, proliferation, and apoptosis; thus, they are considered to be oncogenic drivers or tumor suppressors in various cancers.^[4,7]^

Furthermore, there is an interconnected network of ncRNAs that supports the regulation of physiological activity, of which multiple ncRNAs function as competitive endogenous RNAs (ceRNAs).^[4]^ For instance, some lncRNAs are ceRNAs that can act as miRNA inhibitors (“sponges”), competitively bind and inhibit the expression of miRNAs with tumor-suppressive effects.^[8]^ Perturbation of such interactions may impact cellular activities and lead to carcinogenesis.

Untranslated regions (UTRs) are noncoding fragments at both ends of mRNA molecules and play an important role in the regulation of mRNAs by miRNAs.^[9]^ The 5’ end of mRNA is referred to as the 5’-UTR, while the 3’ end is called the 3’-UTR. Although most properties of proteins are determined by their corresponding mRNA sequences, UTRs possess many binding sites for regulatory signals, many of which are regulatory proteins and miRNAs that influence mRNA translation initiation levels.^[9,10]^ Therefore, their mutations can affect the translation of proteins, which in turn affects cellular activities and initiates oncogenes.^[11]^

Both ncRNAs and UTRs play important regulatory roles in normal cells and maintain normal cellular physiological activity. Their ability to control gene expression may lead them to act as mediators or suppressors of tumorigenesis. In this review, we aim to outline the role and changes in some ncRNAs and UTRs in the development of cancers, in the hope of finding new methods for diagnosis and treatment.

## 2. Methods

We searched PubMed for English-language literature related to ncRNAs and cancers without article type limits, including articles about the role of microRNAs, long ncRNAs, circRNAs, and UTRs in different cancers in the last 10 years, with some necessary supplements (references over the range).

## 3. Results

### 3.1. Role of microRNAs in cancer

MicroRNAs (miRNAs) are small regulatory ncRNAs that contain approximately 18-25 nucleotides.^[12]^ There exists a conserved miRNA region on nucleotides 2-7, which is considered the “seed” and guides miRNA target recognition.^[13]^ The seed sequence at the 5’ end of functional miRNA can form Watson-Crick pairing with the 3’-UTR of the target mRNA to promote targeted mRNA degradation or induce translational silencing.^[14]^ miRNAs act as powerful regulators that target numerous transcripts,^[15]^ and their aberrations may disrupt signaling pathways and physiological activities, leading to cancer progression. They function as oncogenes or tumor suppressors in various cancers and affect the hallmarks of malignant tumors, including programmed cell death, limitless replication potential, sustained angiogenesis, self-sufficiency in growth signals, insensitivity to growth-inhibitory signals, invasion and metastasis.^[16]^ However, the initiation of a tumor or malignancy is not the only result of the action of one type of miRNA. Alterations that lead to cancer characteristics result from many miRNAs acting together.

#### 3.1.1. Mir-34 family.

The miR-34 family contains 3 types, miR-34a, miR-34b, and miR-34a, of which *miR-34b* and *miR-34c* are located on one chromosomal locus.^[17]^ The methylation of these genes leads to silencing. miR-34b/c is more strongly associated with cancer metastasis than miR-34a is. It inhibits anchorage-independent growth and epithelial-to-mesenchymal transition (EMT) in cancer cells, suppressing cancer growth, invasion, and metastasis.^[17]^ However, higher levels of miR-34b/c methylation have been found in nonsmall cell lung cancer (NSCLC) and its low expression level tends to correlate with age, gender, smoking status, tumor progression, poor prognosis and cancer metastasis.^[18]^ Hence, their methylation could be associated with NSCLC development and could be used as a prognostic marker.

**Figure 1.**
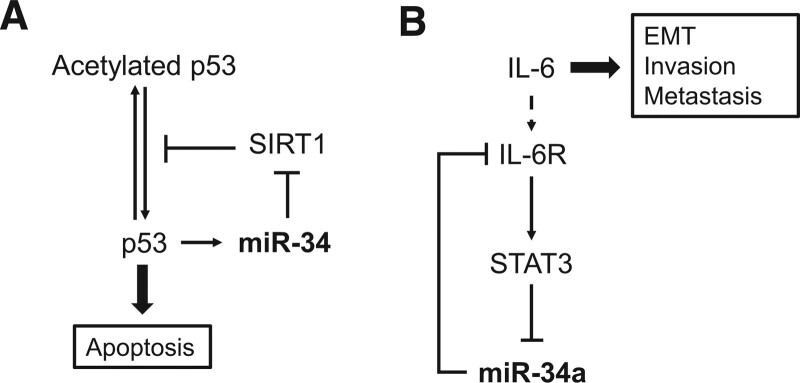
The miR-34 family function as tumor suppressors. (A) Schematic representation of p53/miR34 loop; (B) Schematic representation of IL-6R/STAT3/miR-34a loop. Abbreviations: EMT = epithelial-mesenchymal transition, IL-6 = Interleukin-6, IL-6R = Interleukin-6 receptor, SIRT1 = sirtuin 1, STAT3 = signal transducer and activator of transcription 3.

The loss of miR-34a can be noted in many cancer types, including lung, prostate, and breast cancers,^[19]^ and its reduction mainly aberrates apoptosis. It can target cell cycle-related and antiapoptotic genes to exert anticancer effects. As seen in Figure 1A, as an miRNA family directly regulated by p53, miR-34a involves in the regulation of many oncogenes, the most prominent of which is the regulation of apoptosis.^[20]^ NSCLC exhibits multiple somatic mutations in several genes, among which mutations in the RAS and p53 pathways are important.^[21,22]^ miR-34a can slow cancer progression and increase survival in human KRAS-positive and p53-negative lung cancer patients by repressing Met and BCL-2 expression.^[21]^ This may be assisted by the involvement of spliceosome-associated factor 3 (SART3), a putative pre-miR-34a RNA binding protein (RBP), which is also involved in the miR-34a-CDK4/6 axis for G1 arrest.^[23,24]^

In addition, a study on triple-negative breast cancer (TNBC) found that knockdown of multiple copies of T-cell malignancy 1(MCT-1) can restore the level of miR-34a, which represses IL-6 expression and activates pro-inflammatory M1 macrophage polarization.^[25]^ There exists an IL-6R/signal transducer and activator of transcription 3 (STAT3)/miR-34a loop, in which upregulation of miR-34a can inhibit IL-6-induced EMT, reducing cancer invasiveness and metastasis (Fig. 1B).^[23,25]^ This pathway also functioned in colorectal cancer that miR-34a represses IL-6R expression through a highly conserved miR-34a seed-matching sequence in the IL-6R 3’-UTR. The expression of IL-6 and miR-34a is mutually inhibited, whereas p53 can disrupt this balance by upregulating miR-34a expression.^[23]^ Furthermore, miR-34a can modulate the Notch signaling pathway to accelerate senescence and reduce TNBC cell proliferation and migration.^[26]^

In conclusion, the miR-34a family acts as a tumor suppressor, promotes apoptosis, inhibits metastasis, and is an important target for cancer treatment.

#### 3.1.2. Let-7.

**Figure 2.**
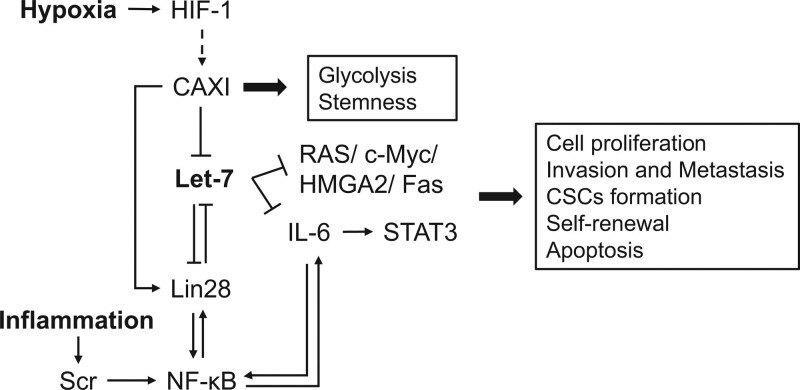
Schematic illustration of let-7/Lin28 axis involvement in inhibition of cancer. Abbreviations: CSCs = cancer stem cells, CAIX = carbonic anhydrase IX, HIF-1 = hypoxia-inducible factor-1, c-Myc = cellular-myelocytomatosis viral oncogene, IL-6 = Interleukin-6, HMGA2 = high mobility group AT-hook 2, NF-κB = nuclear factor kappa-light-chain-enhancer of activated B cells, STAT3 = signal transducer and activator of transcription 3.

In addition to members of the miR-34 family, let-7 is a potent cancer suppressor. Its reduction can be observed in a variety of cancers and is associated with poor prognosis and stemness. The processes of let-7 biosynthesis and expression are interfered with by the Lin28 proteins, which are RBPs containing Lin28A and Lin28B. The expression of let-7 and Lin28 is reciprocally repressed, in that Lin28 represses the expression of let-7, whereas let-7 binds to the 3’-UTR of Lin28 for inhibition.^[27]^ Their expression forms a Lin28/let-7 axis and influences the development of diverse cancers, especially in the process of stemness (Fig. 2).

A study on breast cancer showed that carbonic anhydrase IX (CAIX) can adapt cancer cells to a hypoxic and acidic environment through the Lin28/let-7 pathway, promoting glycolysis and stemness. Silencing or suppressing CAIX can upregulate let-7 and inhibit Lin28.^[28]^ Meanwhile, the increase in let-7 causes a decrease in IL-6, which further suppresses nuclear factor-κB (NF-κB) and STAT3 expression, resulting in diminished proliferation and inflammation.^[28,29]^ A positive inflammatory feedback loop between IL-6 and let-7 has been identified. Inflammation can deliver growth signals, leading to the proliferation of malignant tumor cells.^[29]^ In addition, research has shown that M1 macrophages can induce self-renewal of cancer stem cells (CSCs). Increased expression of LIN-28 and high motility group box protein 2 (HMGA2), and decreased expression of let-7, the LIN-28B-let7-HMGA2 axis engages in the EMT process to accelerate metastasis and induce CSCs.^[30]^ HMGA2 is a target gene for let-7; attenuation of its expression can elevate E-cadherin expression and inhibit fibronectin induced by M1 macrophages, whereas this inhibition can be reverted by HMGA2 3’-UTR translocation.^[31,32]^

In addition to IL-6 and HMGA2, let-7 can target many genes, including RAS, c-Myc, and Fas, which are engaged in self-renewal, differentiation, tumor cell growth, and apoptosis.^[32–34]^ By suppressing let-7 expression, cancer cells exhibit stemness and self-sufficiency in growth signaling. Thus, let-7 is a potent therapeutic target for inhibiting tumorigenesis.

#### 3.1.3. Mir-17-92 cluster.

However, studies have found that a proportion of miRNAs also behave as oncogenes that tend to be upregulated in cancer. They are known as “oncomirs” that often disrupt tumor suppressors or perturb normal cellular signaling pathways, leading to the development of cancer.^[35]^

Hepatitis B virus infection led to a surge in miR-17-92, which further impaired the expression of DEAD-Box helicase 5 (DDX5) by targeting its mRNA 3’-UTR. The reduction of DDX5 facilitated the expression of c-Myc and hepatic cancer stem cell-like genes, aberrant AKT phosphorylation, and AMPK activity, as well as activated Wnt/β-catenin signaling, inducing stemness.^[36]^

In addition, miR-17-92 has been shown to cooperate in cancers, including B-cell lymphoma,^[37,38]^ colorectal cancer,^[39]^ basal cell carcinoma,^[40]^ and lung cancer.^[41]^ Several mechanisms may be involved in the oncogenicity of this cluster, but they all collectively indicate a c-Myc/miR-17-92/PTEN axis that causes carcinogenesis and reduces apoptosis by repressing the PI3K/Akt signaling pathway.^[38–40]^ However, research has reported that miR-17-5p can tightly regulate c-Myc-induced cell proliferation and act as a tumor suppressor.^[42]^ miR-17-92 sensitively coordinates MYC oncogene activity and suppresses E2F by participating in the MYC feedforward loop, leading to cell cycle arrest and repression of tumor cell proliferation.^[43]^ It might be speculated that despite belonging to the same miRNAs, different isoforms might affect cell proliferation and tumorigenesis in a cell type-specific manner, while it might also depend on the function of different target mRNAs.

#### 3.1.4. Mir-210.

miR-210 is also an oncomir that is mainly involved in angiogenesis and metastasis. miR-210-3p consistently activates NF-κB to promote EMT, invasion, and induction of prostate cancer bone metastases. It takes its action through repressing TNF-α-induced protein 3 interacting protein 1 (TNIP1) and suppressor of cytokine signaling 1 (SOCS1), which act as negative regulators and inhibit the NF-κB signaling pathway.^[44]^ A surge in miR-210-3p was found in hypoxic environments,^[44]^ while its disorder could enhance HIF-1α expression and inhibit p53, causing aerobic glycolysis, which further enhances the role of hypoxia as a deleterious factor in carcinogenesis.^[45]^

Furthermore, the overexpression of miR-210 has been shown to be related to angiogenesis. The JAK2/STAT3 and PI3K/Akt pathways may play a major role in cancer angiogenesis,^[46,47]^ especially the latter, which is also implicated in tumor metastasis and invasion.^[48]^

Several miRNAs are involved in various processes of cancer, contributing to the complexity of cancer development and the diversity of its features. Therefore, numerous miRNAs have been investigated as tumor markers or therapeutic targets.

### 3.2. Role of lncRNAs in cancer

Long noncoding RNAs (lncRNAs) are a series of ncRNAs with transcripts longer than 200 nucleotides.^[12]^ Their expression exhibits more tissue-specific properties than that of mRNAs.^[49]^ According to their functions, lncRNAs can be classified as signaling, decoy, guide, or scaffold lncRNAs.^[50]^ Although the exact mechanisms are not yet clear, lncRNAs are involved in the regulation of gene expression and epigenetic modifications and are involved in genomic imprinting, cell differentiation, and inactivation of the X-chromosome.^[49,51]^ Additionally, numerous lncRNAs function as miRNA sponges to compete for expression. Their regulatory roles and their modulation functions in chromatin structures via recruiting chromatin-modifying enzymes, may indicate their importance in pluripotency and gene regulation.^[52]^ Therefore, misexpression of lncRNAs causes cancer development and plays a role in tumor progression. However, the differential expression of lncRNAs in various cancers may suggest potentially important values for diagnosis, treatment, and prognosis.

#### 3.2.1. Hotair.

HOX transcript antisense intergenic RNA (HOTAIR) is a scaffold lncRNA whose expression is related to metastasis and invasion in various cancers. HOTAIR can bind to polycomb repressive complex 2 (PRC2) and lysine-specific demethylase 1A (LSD1) for chromatin modulation.^[53]^ However, its high expression was observed in primary breast carcinomas and was evident in metastatic foci. By targeting PRC2 and mediating histone H3 lysine 27 (H3K27) trimethylation, HOTAIR induces PRC2 occupancy and alters many genes involved in cell-cell signaling and development pathways, leading to tumor metastasis, invasion, and angiogenesis.^[54]^ Its ability to induce breast cancer growth and metastasis was also demonstrated by Ren *et al*^[55]^ that elevated HOTAIR expression facilitated EMT and tumor metastasis.

Furthermore, upregulation of HOTAIR attenuated cell apoptosis induced by TNF-related apoptosis-inducing ligand (TRAIL) in pancreatic cancer. Under the involvement of enhancer of zeste homolog 2 (EZH2), HOTAIR suppressed death receptor 5 (DR5) expression, which is a TRAIL receptor, resulting in resistance to TRAIL-induced apoptosis in pancreatic cancer.^[56]^ Overexpression of HOTAIR is accompanied by overexpression of hexokinase-2 (HK2), resulting in accelerated energy metabolism in pancreatic adenocarcinoma cells.^[57]^

An increase in HOTAIR was also detected in NSCLC and paralleled by a decrease in miR-34a-5p, a tumor suppressor miRNA. Altering their interaction through the HOTAIR/miR-34a-5p axis could suppress Snail expression, thereby increasing E-cadherin levels and repressing EMT.^[20]^ Consistently, similar expression was found in esophageal cancer and PDAC, where HOTAIR acts as a competing endogenous lncRNA (ceRNA) to accelerate tumorigenesis by sponging miRNA.^[58,59]^

Interfering with the interaction of HOTAIR with other molecules and the HOTAIR/miRNA axis has therapeutic implications and may be sensitive to target tumors overexpressing HOTAIR.

#### 3.2.2. MALAT1.

Metastasis-associated lung adenocarcinoma transcript 1 (MALAT1) can regulate the transcriptional processes of multiple genes and plays a role in a wide range of biological pathways, including glycolysis, adhesion, DNA repair, and vascularization, but it is chiefly involved in alternative splicing.^[60]^

Notably, its expression is known to be a predictive marker for lung cancer, particularly metastasis.^[60]^ When MALAT1 is knocked down in A549 cells, dysregulation of metastasis-associated transcripts and growth control genes can be observed in lung cancer cells.^[61]^ In addition, silencing MALAT1 restored the expression level of E-cadherin and inhibited the EMT process, thereby reducing brain metastases in patients with lung cancer.^[62]^ While similar results were not observed in HeLa cells, implying that the role of MALAT1 may be cell type-specific.^[61]^

Patients with osteosarcoma (OS) with high levels of MALAT1 expression exhibit a poor prognosis, a high rate of cancer metastasis, and an impact on tumor stage and size. The high metastasis rate induced by MALAT1 is associated with agonism of the PI3K/AKT pathway and reduced E-cadherin expression via the involvement of EZH2.^[63]^ Furthermore, MALAT1 can act as an miRNA sponge to regulate the miR-34a/cyclin D1 and miR-206/CDK9 axes, promoting OS tumor cell viability, proliferation, and activating tumor progression.^[63,64]^

Similarly, its role as a ceRNA also occurs in colon cancer. By sponging miR-129-5p, MALAT1 enhances colon tumorigenesis and increases the expression of high mobility group box protein 1 (HMGB1), a target of miR-129-5p that ordinarily protects cells from injury.^[65]^ Analogous findings exist in the MALAT1/miR-429 axis in cervical cancer,^[66]^ MALAT1/miR-204/IGF2BP2/m6A-MYC axis in thyroid cancer,^[67]^ MALAT1/miR-23b-3p/ATG12 axis in gastric cancer (GC),^[68]^ and MALAT1/miR-140/BIRC6 axis in prostate cancer.^[69]^ Fortunately, knockdown and silencing can reverse the effects of MALAT1, inhibit the growth and metastasis of tumor cells, and promote apoptosis.

Although MALAT1 has been shown to be expressed in many cancers to promote cancer development and metastasis, Kim *et al* reported that its overexpression in breast cancer inhibits tumor metastasis. However, there is a lack of convincing evidence.^[70]^

#### 3.2..3. H19.

H19 is thought to be an oncofetal lncRNA associated with several cancers. Its upregulation can be seen in the hypoxic stress response via the p53/HIF1-α pathway and induces cell proliferation.^[71]^ Furthermore, H19 overexpression upregulates Lin28 by repressing let-7, leading to invasion and metastasis in breast cancer. It has been demonstrated that by modulating the H19/let-7/Lin28 network, autophagy can be enhanced by the inhibition of the EMT process.^[72]^

In colorectal cancer (CRC), H19 promotes the EMT process and causes CRC lung metastasis by functioning as a sponge of miR-22-3p and miR-29b-3p, increasing the expression of matrix metalloprotease-14 (MMP14) and progranulin (PGRN), which further acts on the Wnt/β-catenin signaling pathway to enhance EMT.^[73,74]^ In addition, by inhibiting miR-141, H19 can activate the β-catenin signaling pathway, promoting CSC stemness and chemoresistance.^[75]^ H19 is also involved in stemness through a positive feedback loop in thyroid carcinomas, where estradiol upregulates H19 expression via estrogen receptor β (ERβ) and H19 functions as an miRNA-3126-5p sponge to induce Erβ expression in turn.^[76]^

#### 3.2.4. NKILA.

Surprisingly, some lncRNAs play a role in cancer suppression. NF-κB-interacting lncRNA (NKILA) has been identified as a tumor suppressor that regulates NF-κB activity, and its expression is decreased in various cancers.^[77]^

A lncRNA-NKILA/NF-κB feedback loop exists in which NKILA can firmly combine with the NF-κB: IκB complex to inhibit the phosphorylation of IκB, leading to NF-κB inactivation and inflammation suppression, whereas NF-κB can bind to the NKILA promoter region and initiate its transcription.^[78,79]^ Herein, Wu *et al* also confirmed that TGF-β upregulated NKILA expression in an NF-κB-dependent manner, which in turn regulated NF-κB activity and EMT in breast cancer. In addition, there is a negative feedback mechanism whereby NKILA suppresses TGF-β-mediated EMT by inhibiting NF-κB.^[80]^ In addition, lower levels of NKILA lead to the activation of IκBα phosphorylation and the NF-κB pathway in rectal cancer, causing tumor proliferation and metastasis, ultimately resulting in exacerbation of the clinical stage of the tumor and poor prognosis.^[81]^

It can be speculated that the induction of NKILA expression could be a therapeutic approach for cancer. NKILA overexpression attenuates radiation resistance in laryngeal cancer cells.^[78]^ Nevertheless, it has been reported that NKILA assists tumor cells to avoid immunosurveillance. With the involvement of the NF-κB signaling pathway, high expression of NKILA can sensitize activated T cells to tumor-induced activation-induced cell death (AICD), resulting in cytotoxic T lymphocyte death and immunological disorders.^[82]^ From this perspective, inhibition of NKILA expression has a facilitative effect on the efficacy of these approaches for treating cancer by immunological means.

In brief, most lncRNAs act primarily as oncogenic factors in tumor progression, whereas some have tumor-suppressive effects (Table 1). By sponging miRNAs with cancer-suppressive effects and promoting the expression of their downstream targets, they can disturb biological and cellular processes. There seems to be RNA crosstalk involving lncRNAs, miRNAs, mRNAs, or proteins to form a ceRNA network and functions in cancer development.^[83]^

**Table 1:** Expression and mechanisms of some long noncoding RNAs in cancers.

lncRNA	Cancer type	Expression	Mechanism	Results	Ref.
HOTAIR	Breast cancer	↑	HOTAIR targeted PRC2	Metastasis and invasion↑; Cancer aggression↑; Cell growth↑; Angiogenesis↑	^[54]^
HOTAIR	Breast cancer	↑	TGF-β1/HOTAIR axis	Expression of E-cadherin↓; Expression of vimentin and β -catenin↑; EMT↑; Metastasis↑; Cell growth↑; Drug resistance↑	^[55]^
HOTAIR	Pancreatic cancer	↑	HOTAIR regulated DR5 expression via EZH2	TRAIL resistance↑; Apoptosis↓; Cell invasion and proliferation↑	^[56]^
HOTAIR	Pancreatic cancer	↑	HOTAIR promoted HK2 expression	Cell proliferation↑; Lactate production↑; Glucose uptake↑; ATP production↑; Cancer energy metabolism↑	^[57]^
HOTAIR	Lung cancer	↑	HOTAIR/miR-34a-5p axis	miR-34a-5p expression↓; Migration and invasion↑; Tumorigenesis↑; Cell proliferation and growth↑; Expression of E-cadherin↓; Expression of vimentin and snail↑; EMT↑; Poor prognosis	^[20]^
HOTAIR	Esophageal cancer	↑	HOTAIR/miR-148a axis	miR-148a expression↓; Expression of Snail2↑; C proliferation, growth and differentiation↑;Invasion and metastasis↑; EMT↑	^[58]^
HOTAIR	Pancreatic cancer	↑	HOTAIR and EZH2 inhibited miRNA-34a	miR-34a expression↓; Cell proliferation and growth↑	^[59]^
MALAT1	Lung cancer	↑	MALAT1 regulates several metastasis-related genes expression	Cell metastasis and extravasation↑	^[61]^
MALAT1	Lung cancer	↑	MALAT1 enhanced gene expression of cell motility and metastasis	Brain metastasis↑; EMT↑; Invasion and metastasis↑; Cancer cell motility and migration↑; E-cadherin expression↓; Patients’ survival rate↓	^[62]^
MALAT1	Osteosarcoma	↑	MALAT1/miR-34a/cyclin D1 axis	miR-34a expression↓; Cyclin D1 expression↑; OS cell viability and growth↑; Tumor invasion and migration↑; Tumor size, clinical stage and distant metastasis in patients↑	^[63]^
MALAT1	Osteosarcoma	↑	MALAT1/miR-206/CDK9 axis	miR-206 expression↓; CDK9 expression↑; OS cell proliferation↑; OS progression↑; Apoptosis↓	^[64]^
MALAT1	Colon cancer (colorectal cancer)	↑	MALAT1/miR-129-5p/HMGB1 axis	miR-129-5p expression↓; HMGB1 expression↑; Cell proliferation↑; Cancer progression↑	^[65]^
MALAT1	Cervical cancer	↑	MALAT1/miR-429 axis	miR-429 expression↓; Cervical cell viability and proliferation↑; Apoptosis↓; Cell invasion↑	^[66]^
MALAT1	Thyroid cancer	↑	MALAT1/ miR-204/IGF2BP2/m6A-MYC axis	miR-204 expression↓; Expression of IGF2BP2 and MYC↑; Cell proliferation↑; Migration and invasion↑; Apoptosis↓;	^[67]^
MALAT1	Gastric cancer	↑	MALAT1/miR-23b-3p/ATG12 axis	miR-23b-3p expression↓; ATG12 expression↑; Chemoresistance↑; Autophagy↑	^[68]^
MALAT1	Prostate cancer	↑	MALAT1/miR-140/BIRC6 axis	miR-140 expression↓; BIRC6 expression↑; Cell proliferation↑; Migration and invasion↑; Tumor growth↑; Apoptosis↓	^[69]^
MALAT1	Breast cancer	↑	MALAT1 inactivated TEAD	Lung metastasis↓	^[70]^
H19	Breast cancer	↑	H19/let-7/Lin28 loop	let-7 expression↓; Lin28 expression↑; EMT↑; Invasion and metastasis↑; Expression of autophagy-associated molecules beclin-1 and LC3-II↓;	^[72]^
H19	Colon cancer (colorectal cancer)	↑	H19/miR-22-3P/MMP14 axis	miR-22-3p expression↓; MMP14 expression↑; Metastasis↑; EMT↑; Expression of E-cadherin↓; Expression of fibronectin and ITGA5↑	^[73]^
H19	Colon cancer (colorectal cancer)	↑	H19/miR-29b-3p/PGRN axis	miR-29b-3p expression↓; PGRN, β-catenin and Wnt expression↑; Expression of E-cadherin↓; Expression of vimentin, snail, c-Myc and cyclin D1↑; Cell proliferation↑; Metastasis↑	^[74]^
H19	Colon cancer (colorectal cancer)	↑	H19/miR-141/β-catenin axis	miR-141 expression↓; β-catenin expression↑; Tumor stemness and chemoresistance↑; Tumor development↑	^[75]^
H19	Thyroid cancer	↑	ERβ-H19 positive feedback loop	miRNA-3126-5p expression↓; Expression of ERβ↑; Stemness↑	^[76]^
NKILA	Laryngeal cancer	↓	lncRNA-NKILA/NF-κB feedback loop	NF-κB signaling activation; Cell proliferation, viability↑; Cell migration↑; Invasion↑;Radio resistance↑	^[78]^
NKILA	Breast cancer	↓	lncRNA-NKILA/NF-κB feedback loop	IkB phosphorylation↑; Metastasis↑; Cancer progression↑; Poor prognosis; Inflammation↑	^[79]^
NKILA	Breast cancer	↓	NKILA inhibits TGF-β-induced EMT by suppressing NF-κB	Expression of NF-κB and TGF-β↑; EMT↑; Invasion and metastasis↑; Expression of snail and vimentin↑; E-cadherin expression↓	^[80]^
NKILA	Rectal cancer	↓	lncRNA-NKILA/NF-κB feedback loop	NF-κB pathway activation; Cell proliferation↑; Invasion and metastasis↑; Poor prognosis	^[81]^
NKILA	Breast and lung cancer	Expression in activated T cell ↑	Activation of NF-κB signaling pathway	AICD activation; CTLs and T_H_1 death↑; Tumor immune evasion↑; Patient’s survival↓	^[82]^

AICD = activation-induced cell death, ATG12 = autophagy-related 12, BIRC6 = baculoviral IAP repeat-containing 6, CDK = cyclin-dependent kinases, CTLs = cytotoxic T lymphocytes, DR5 = death receptor 5, EMT = epithelial-mesenchymal transition, Erβ = estrogen receptor β, EZH2 = enhancer of zeste homolog 2, HK2 = hexokinase-2, HMGB1 = high motility group box protein 1, HOTAIR = HOX transcript antisense intergenic RNA, IGF2BP2 = insulin-like growth factor 2 mRNA binding protein 2, ITGA5 = integrin subunit alpha 5, LC3-II = microtubule-associated protein light chain 3 II, LncRNA = long noncoding RNA, MALAT1 = metastasis associated lung adenocarcinoma transcript 1, MMP14 = matrix metalloprotease-14, MYC = myelocytomatosis, NKILA = NF-κB-interacting lncRNA, NF-κB = nuclear factor kappa-light-chain-enhancer of activated B cells, OS = osteosarcoma, PGRN = progranulin, PRC2 = polycomb repressive complex 2, TEAD = TEA domain family member, TRAIL = TNF-related apoptosis-inducing ligand, TGF-β = transforming growth factor beta, T_H_1 = type 1 helper T cells.

### 3.3. Role of circRNAs in cancer

Circular RNAs (circRNAs) are unique compared to other ncRNAs in that their 5’ and 3’ ends are linked end-to-end to form a closed loop. They can directly target the UTRs of mRNAs for gene regulation.^[84]^

circRNAs tend to be highly expressed in terminally differentiated or proliferation-stable cells.^[85]^ However, some circRNAs paradoxically show high expression in cancer, causing cancer progression. These circRNAs can function as miRNA sponges to inhibit their expression. In NSCLC, circ-ZKSCAN1 acts as a miR-330-5p sponge and influences downstream mRNA expression to inhibit the MAPK signaling pathway.^[86]^ Furthermore, circ-CPA4 is highly expressed in NSCLC cells and represses let-7 expression. Through the circ-CPA4/let-7 axis, circ-CPA4 can further enhance programmed cell death ligand 1 (PD-L1), a target of let-7 that induces cell stemness and drug resistance.^[87]^ A similar mechanism of circRNAs as ceRNAs in promoting carcinogenesis has been found in other cancers, including breast,^[88,89]^ colorectal,^[90]^ endometrial cancer,^[91]^ and esophageal squamous cell carcinomas.^[92]^

On top of serving as an miRNA sponge, it can also regulate downstream gene targets to promote cancer cell growth and invasion. circ0005276 can positively target X-linked inhibitor of apoptosis protein (XIAP), enhance EMT, and induce prostate cancer progression.^[93]^

In contrast, some circRNAs with reduced expression levels exhibited a suppressive effect on cancer cells. Once again, circ-ZKSCAN1 can inhibit the transcriptional actions of the Wnt/β-catenin signaling pathway by blocking another target, fragile X mental retardation protein (FMRP), suppressing the process of stemness.^[94]^ Furthermore, hsa_circ_100395 can act as an miRNA sponge to inhibit miR-1228, which acts as an oncogene in lung cancer, and represses p53 expression to inhibit apoptosis.^[95,96]^ We can infer that different circRNAs, or even for the same circRNA, their promotive or inhibitory effects on cancer are influenced by their targets and cell-specificity.

### 3.4. Role of UTRs in cancer

UTRs can impact proteins by managing the translation and location of mRNAs and causing mRNA degradation, thereby determining the fate of proteins by modulating protein-protein interactions (PPIs).^[97]^ The 3’-UTR acts as an intermediate regulator of mRNA to specific RNA-binding factors. Multiple factors can act collaboratively or competitively on the same mRNA 3’-UTR, eliciting final effects influenced by their respective expression levels, binding sites, and cellular activity.^[98]^ Thus, disturbance of the UTR can result in tumorigenesis.

As it has briefly mentioned the presence of a MALAT1/ miR-204/IGF2BP2/m6A-*MYC* axis above, the insulin-like growth factor-2 mRNA-binding proteins 2 (IGF2BP2, IMP-2) belongs to the IGF-2 mRNA-binding proteins 1,2 and 3 (IMPs) family, which is a tumor promoter can enhance tumor spread.^[99]^ It targets the 3’-UTR region of cyclin D1, D3, and G1 mRNA, and with a surge in their expression, the cell cycle proceeds to cause cancer cell proliferation.^[100]^ When IMP acts on the 3’-UTR of the 5.0kb CD44 mRNA, IMP contributes to CD44 stabilization, which further reduces cell adhesion, generates invadopodia, and ultimately promotes cancer cell metastasis.^[99]^

There is an AU-rich element (ARE) in the 3’-UTR, which is predominantly expressed in genes that require rigorous regulation and lead to rapid mRNA disassembly. ARE does not directly affect protein abundance, but can influence the gene expression levels of protooncogenes and cytokines by binding to RBPs or miRNAs. Therefore, alterations in AREs or disruption of ARE-containing mRNAs can lead to cancer development.^[97,101]^ Precisely, this is supported by the finding that cancer cells typically express many mRNA isoforms with short 3’-UTRs lacking the ARE parts caused by alternative polyadenylation (APA).^[11,102,103]^ The shorter 3’-UTR was observed in hematopoietic- and neurologic-expressed sequence 1 (HN1) in carcinomas. In contrast, when HN1 has a longer 3’-UTR, it is usually less stable and maintains a lower expression level, which can cause cellular senescence and improve patient survival.^[103]^ Therefore, it is noteworthy that APA-induced shortening of 3’-UTRs is a mechanism that causes cancer development.

In addition, 5’-UTRs also participate in carcinogenesis. A study on hepatocellular carcinoma found that *YTHDF2-OCT4* signaling is involved in cancer progression. By targeting m6A in the 5’ UTR of *OCT4* mRNA, *YTHDF2* enhanced the methylation of m6A and upregulated OCT4 expression, a pluripotency factor whose enhanced expression increased the CSC phenotype and lung metastasis.^[104]^

Furthermore, some reports have suggested that the translation of p53 can be regulated through its 5’ or 3’-UTR, especially the 5’ UTR of p53 mRNA, which can regulate the expression of p53 by binding to the 5’ UTR of its mRNA.^[105,106]^ There are two internal ribosome entry sites (IRESs) in p53 mRNA, with one located in the 5’-UTR region, which regulates p53 translation when DNA is damaged. However, this abnormality was found in oncogene-induced senescence, leading to an increase in p53 translation.^[107]^ p53 itself can negatively regulate its translation by acting on the 5’-UTR of p53 mRNA.^[108]^ Strikingly, lncRNAs also participate in the regulation of p53, and there is a complicated network between them.^[109]^

The combination of all these complexities and the binding and interaction of UTRs with proteins and ncRNAs literally complicates their role in cancers.

### 3.5. NcRNA network and therapy

**Figure 3.**
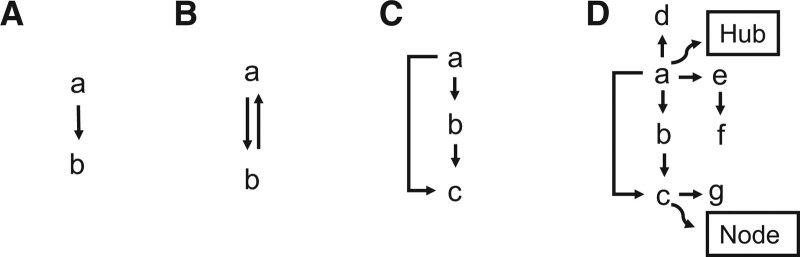
Representation of motifs in networks. (A) Simple regulation. (B) Feedback loop. (C) Feedforward loop. (D) Simplified network motifs. Examples of nodes and hubs are noted. *The arrows here do not represent facilitation but only the direction of action.

It is difficult to identify ncRNA functions in isolation; many ncRNAs exhibit highly complex interactions in cancer progression and regulate the cellular growth cycle and properties. A vast network of intersecting ncRNAs passes different signals from one level to the next and many ncRNA interactions correspond to characteristic patterns and are part of network motifs.^[110]^ Interactions between ncRNAs and ncRNAs, genes, other transcriptomes, or proteins are not only one-way regulation, but can also be feedback or feedforward regulation (Fig. 3). The aforementioned positive feedback loops between let-7 and IL-6 or the interaction between H19 and ERβ are both feedforward regulators. Compared to feedforward loops, negative feedback loops are more common in ncRNA networks and play an important role in maintaining inputs and outputs within a normal range.^[4]^ These simple and fragmented parts intertwine and intersect like rivers, forming complex networks. Among them, the most representative is the competitive endogenous RNA (ceRNA) networks.^[5]^

As we have already mentioned that some ceRNAs, which means that these ncRNAs are in competitive regulatory interactions, and generally function as miRNA sponges to suppress their functions. For example, in the case of the H19/let-7/Lin28 axis, H19 inhibits the expression of let-7, elevating Lin28 levels, which are normally inhibited by let-7, to promote EMT and inhibit cellular autophagy.^[72]^ ncRNAs include lncRNAs, pseudogenes, circRNAs, or even mRNAs, and 3-UTRs can act as ceRNAs in cancers and have the potential to initiate tumorigenesis.^[111,112]^ They can decoy miRNAs, attenuate their regulation of genes, transcripts, or proteins, and serve as posttranscriptional regulators. Precisely because of their interaction with each other, once one of the components has changed, the impact can be far-reaching and perturbation can contribute to disease pathogenesis, including carcinogenesis.

Therefore, it has become a major research topic in diagnosis and prognosis. Studies have focused on ceRNA interactions to identify disordered RNA expression in specific cancer types as biomarkers and treatment targets.^[83]^ Tang *et al*^[113]^ found that circ-KIAA1244c was expressed at lower levels in the plasma of GC patients than in healthy subjects, and that the reduced levels were negatively correlated with TNM stage and positively correlated with survival rate. Thus, GC tissue-derived circ-KIAA1244 can be used as a biomarker for the early diagnosis of GC and assist in cancer staging. In addition, with C1632, an inhibitor of Lin28, let-7 expression is elevated, leading to PD-L1 inhibition and enhanced immunity to cancer. LIN28/let-7 therefore also serves as a candidate site for PD-L1-mediated immunotherapy.^[114]^

Presumably, in the absence of a direct action on the target, it may be possible to use their interactions to hold them together for therapeutic purposes. Also, through gene knockdown or silencing, the expression of oncogenic ncRNAs can be inhibited to suppress cancer development.

## 4. Conclusions and Perspectives

As a momentous part of gene regulation, ncRNAs and UTRs can act as oncogenes or tumor suppressors to influence cancer development and affect cancer invasion, metastasis, proliferation, apoptosis, and angiogenesis. They constitute an ncRNA network that plays an important role in cellular life activities and disease development, of which ceRNAs, as competing miRNA decoys, account for a large proportion. Nodes and hubs in the ncRNA network are significant research targets for cancer markers and diagnostics (Fig. 3). Many drug developments act on their interconnections with each other and represent a new direction in cancer therapy.

However, there are some obstacles to ncRNA therapy. First, the key point of ncRNA therapy is to deliver the therapeutic drug to the target cells effectively and induce specific inhibition of the target mRNA while not interfering with the normal mRNA activity.^[115]^ Because RNA interactions are a complex web, targeted drugs must not only be able to reach the target cells and cross heavy cellular structures but also be sufficiently mRNA-specific. Beyond that, it is necessary to find the traffic hub on this network, not just a tiny node. In other words, the drug should act as a powerful target that can inhibit cancer progression rather than just one characteristic of cancer that can lead to possible drug resistance.

## Author contributions

Y.Z. contributed to this work, wrote the paper, and curated the data. M.Y. revised the paper. F.H. and S.Y. contributed equally to this work; they were responsible for the idea, funding, and revision. All the authors have read and agreed to the published version of the manuscript.
